# TAF-4 is required for the life extension of isp-1, clk-1 and tpk-1 Mit mutants

**DOI:** 10.18632/aging.100604

**Published:** 2013-10-04

**Authors:** Maruf H. Khan, Melissa Ligon, Lauren R. Hussey, Bryce Hufnal, Robert Farber, Erin Munkácsy, Amanda Rodriguez, Andy Dillow, Erynn Kahlig, Shane L. Rea

**Affiliations:** ^1^ Barshop Institute for Longevity and Aging Studies and Department of Physiology, University of Texas Health Science Center at San Antonio, San Antonio, TX 78245, USA; ^2^ Institute for Behavioral Genetics, University of Colorado at Boulder, Boulder, CO 80309, USA

**Keywords:** Mit mutants, C. elegans, mitochondria, lifespan, isp-1, clk-1, taf-4, hif-1, aha-1, ceh-18, jun-1, nhr-27, nhr-49

## Abstract

While numerous life-extending manipulations have been discovered in the nematode Caenorhabditis elegans, one that remains most enigmatic is disruption of oxidative phosphorylation. In order to unravel how such an ostensibly deleterious manipulation can extend lifespan, we sought to identify the ensemble of nuclear transcription factors that are activated in response to defective mitochondrial electron transport chain (ETC) function. Using a feeding RNAi approach, we targeted over 400 transcription factors and identified 15 that, when reduced in function, reproducibly and differentially altered the development, stress response, and/or fecundity of isp-1(qm150) Mit mutants relative to wild-type animals. Seven of these transcription factors – AHA-1, CEH-18, HIF-1, JUN-1, NHR-27, NHR-49 and the CREB homolog-1 (CRH-1)-interacting protein TAF-4 – were also essential for isp-1 life extension. When we tested the involvement of these seven transcription factors in the life extension of two other Mit mutants, namely clk-1(qm30) and tpk-1(qm162), TAF-4 and HIF-1 were consistently required. Our findings suggest that the Mit phenotype is under the control of multiple transcriptional responses, and that TAF-4 and HIF-1 may be part of a general signaling axis that specifies Mit mutant life extension.

## INTRODUCTION

In humans, many mutations that disrupt the mitochondrial electron transport chain (ETC) result in pathogenesis; several are the cause of debilitating childhood illnesses [1]. Altered mitochondrial function has also been implicated in various late-onset diseases, including Alzheimer's, Parkinson's and Huntington's Diseases [2]. Unexpectedly, mutations that reduce mitochondrial ETC function can sometimes extend life rather than shorten it. In the nematode *Caenorhabditis elegans*, for example, when any of several components of the ETC are disrupted, either by mutation or RNAi-treatment, lifespan is increased anywhere from 10% to 3-fold over wild-type [3]. RNAi knockdown of subunits in complex I, III, IV or V can extend the lifespan of *Drosophila melanogaster* as well [4]. In the yeast *Saccharomyces cerevisiae*, several mutations that reduce mitochondrial respiration are also associated with increased replicative lifespan, a surrogate measure of mother cell longevity [5]. Finally, in *Mus musculus*, removal of *Surf1* (which encodes a cytochrome c oxidase assembly factor [6]), or hemizygous removal of *Mclk1* (which encodes demethoxyubiquinone monoxygenase, a gene essential for ubiquinone biosynthesis[7]), results in longer-lived animals. Thus, multiple organisms from phyla separated by millions of years of evolution, all respond to some forms of mitochondrial ETC dysfunction with increased lifespan.

Electron transport chain disruptants of *C. elegans* that have extended lifespan are collectively called Mit (Mitochondrial) mutants [8]. While most of the 40+ Mit mutants have been identified via large-scale RNAi screening [9], a handful of *bona fide* mutants have been identified in chemical screens, including the missense or nonsense alleles of *isp-1(qm150), clk-1(e2519* and *qm30)*, and *tpk-1(qm162)*. These genes encode the Rieske iron-sulfur protein [10], the worm ortholog of DMQ monoxygenase [11], and thiamine pyrophosphokinase (TPK-1) [12], respectively. Loss of TPK-1 disrupts cellular thiamine levels, which in turn disrupts the α-ketoacid dehydrogenases that operate the mitochondrial tricarboxylic acid cycle that feeds electrons into the ETC [12, 13]. Recently, we presented evidence suggesting that inhibition of the α-ketoacid dehydrogenases (namely pyruvate dehydrogenase, α-ketoglutarate dehydrogenase and branched-chain α-ketoacid dehydrogenase) may be the proximal mechanism leading to the characteristic Mit phenotype [13]. The downstream molecular players that permit Mit mutants and their defective mitochondria to increase lifespan remain, however, largely unresolved.

In humans, it is well established that many mutations that disrupt the mitochondrial ETC manifest as threshold effect disorders [8]. That is, the phenotypic appearance of the diseased state is not linearly-dependent upon the degree of ETC inhibition caused by the mutation. Compensatory mechanisms, known as retrograde responses, act to delay, or in extreme cases even prevent, disease manifestation. The same is true in the *C. elegans* Mit mutants [3, 14]. A novel retrograde-like response was recently delineated in *C. elegans* by Haynes and colleagues. Under normal conditions, the transcription factor ATFS-1 is imported into mitochondria and degraded. Following mitochondrial dysfunction, cytoplasmically-localized ATFS-1 is instead trafficked to the nucleus whereupon it directly activates a suite of some 391 genes [15]. Loss of ATFS-1 is lethal to both *isp-1(qm150)* and *clk-1(qm30)* Mit mutants. Five additional transcription factors are known to be required for Mit mutant life extension. These are p53/*cep-1*[14], hypoxia factor-1 (HIF-1) [16], the *C.elegans-*specific homeodomain protein CEH-23 [17], the E-twenty-six transcription factor ETS-9 [17], and the nuclear hormone receptor, NHR-25 [17]. A sixth transcription factor, CREB homolog-1 (CRH-1), is almost certainly required as well, given that *aak-2/*AMP kinase (AMPK) is essential for the life extension of *isp-1(qm150)* and *clk-1(qm30)* Mit mutants [18] and for over-expressed AMPK to extend lifespan it must phosphorylate CRH-1 [19]. It is clear, from all these studies, that the life extending mechanisms that are invoked following mitochondrial ETC dysfunction in *C. elegans* involves coordination between multiple pathways. In this study, we now extend these findings by undertaking a screen of worm transcription factors. We establish TAF-4 as a novel transcription factor that mediates the lifespan extension in three distinct Mit mutants: *isp-1, tpk-1* and *clk-1*.

## RESULTS

The Mit phenotype is defined by the concordant appearance of several traits that co-segregate with life extension; these include slowed larval development, reduced adult size, and diminished fertility [3]. We and others have also observed that many stress-response genes, including *hsp-6* [15] and *gst-4* [21] (markers of UPR^mt^ activation and oxidative stress, respectively), are constitutively activated in Mit mutants. We constructed an *isp-1(qm150); Pgst-4::GFP* transcriptional reporter strain and observed that GFP expression was robustly upregulated in the hypodermis and neurons of these animals at the L4/YA boundary – a time when mitochondria are rapidly expanding [23]. GFP expression slowly subsided in these animals as they aged until it reached a level indistinguishable from that in control *Pgst-4::GFP* animals, which contain a faint, albeit constitutive and SKN-1 independent, background hypodermal signal [21].

### Fifteen transcription factors control multiple aspects of the Mit phenotype in *isp-1(qm150)* mutants

To expedite discovery of transcription factors required for Mit mutant life extension we monitored several surrogate phenotypes that co-segregate with increased lifespan in *isp-1(qm150); Pgst-4::GFP* mutants. Phenotypes that were scored include (i) rate of larval development, (ii) viability, (iii) GFP reporter-gene fluorescence, (iv) gross morphology, (v) fertility and (vi) fecundity. We screened over 400 bacterial feeding RNAi clones targeting approximately 40% of the known and predicted transcription factors in *C. elegans* [24] (Supplementary Table II). After three rounds of screening, twenty-four RNAi clones were isolated that differentially altered at least one longevity co-segregating phenotype in *isp-1(qm150); Pgst-4::GFP* mutants relative to control *Pgst-4::GFP* animals. To address possible differences in the efficacy of RNAi between the two strains, and to be certain that the qualitative phenotypes that we used to identify the set of twenty-four RNAi clones were also robust across investigator, we tested if the F_1_ progeny from each strain continued to show the same differential responses as their parents when further cultured on RNAi lawns. Fifteen RNAi clones passed an additional three rounds of F_1_ screening when scored by an independent investigator. The differential effects caused by each RNAi on the two strains are summarized in Table I (refer also to Supplementary Figure S1).

**Figure 1:**
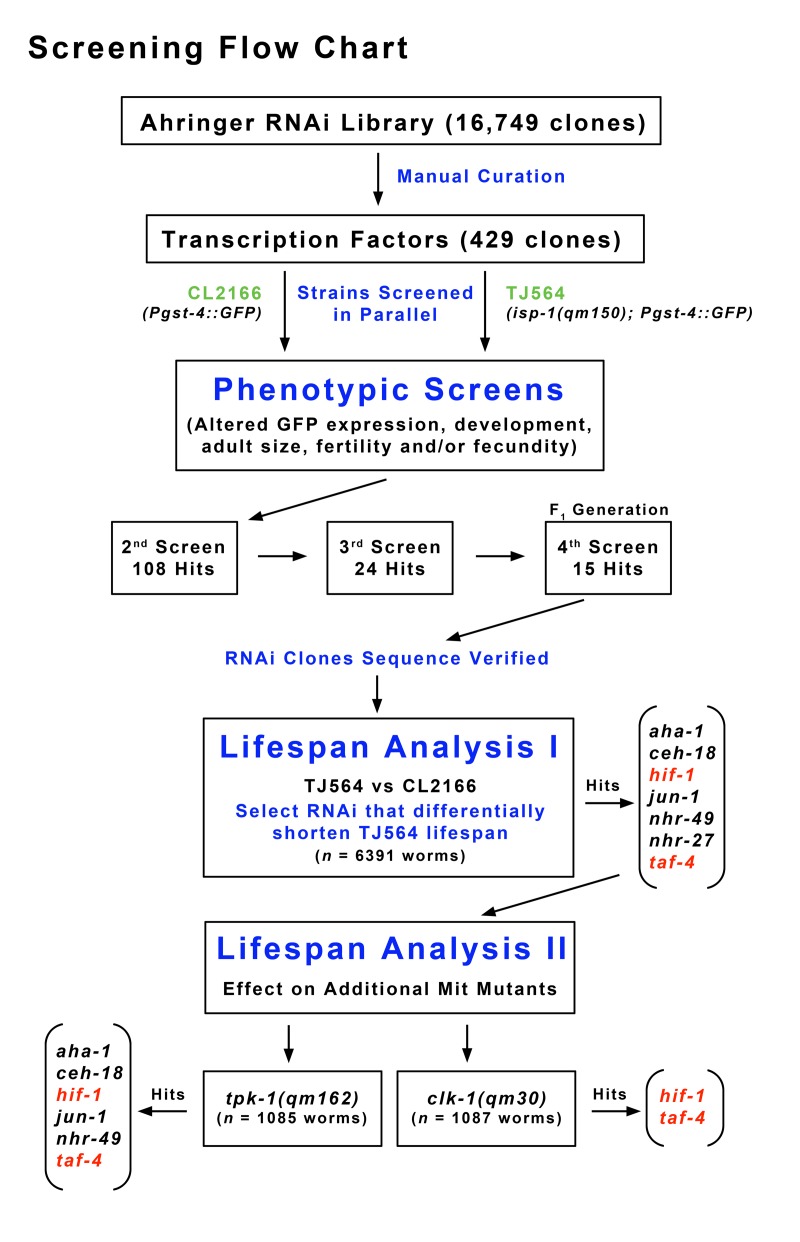
Flow Chart Describing Screening Methodology Feeding RNAi clones corresponding to more than 400 transcription factors were retrieved from the Ahringer RNAi library. *isp-1*; *Pgst-4::GFP* double mutants (TJ564) and a *Pgst-4::GFP* control strain (CL2166), both containing GFP under the control of an oxidative stress-sensitive promoter, were screened in parallel for differential changes in reporter expression, development, adult size, fecundity and fertility. Fifteen transcription factors were identified that reproducibly and differentially altered some aspect of the *isp-1* phenotype relative to the control strain (Table I). Seven of the identified RNAi clones significantly reduced *isp-1* lifespan. Of these only two clones, targeting *taf-4* and *hif-1*, significantly and differentially reduced the lifespan of *isp-1, clk-1* and *tpk-1* Mit mutants relative to control strains.

**Table 1:** Transciption Factor RNAi Clones That Differentially Effect Lifespan Co-segregating Phenotypes of isp-1(qm150)

RNAi	CL2166 Phenotype (vs vector control*)	TJ564 Phenotype (vs vector control)
***aha-1***	Most vector-like, some slow to develop	Most vector-like, some slow to develop and show weak GFP induction
***ceh-18***	Severe developmental asynchrony, fewer F1	Severe developmental asynchrony, almost sterile
***ceh-24***	Vector-like	Medium GFP induction
daf-2**	Vector-like	Thin adults, almost infertile, many abandoned plate
***dpr-1***	Vector-like	Almost infertile, GFP weakly induced
***hif-1***	GFP weakly induced	GFP strongly induced
***jun-1***	Vector-like	Severe developmental asynchrony, sterile adults, some L4 arrest, GFP variably induced
***elt-3***	GFP-suppressed in head hypodermis***	Reduced fertility, medium/strong GFP suppression
***nhr-105***	GFP-suppressed in head hypodermis***	Medium GFP suppression
***nhr-125***	Vector-like	Mild developmental asynchrony, adults weakly induce GFP
***nhr-214***	Vector-like	Mild developmental asynchrony (potential L3/L4 arrest)
***nhr-27***	Vector-like	Reduced fertility (mild), weak/medium GFP induction, F1 pale
***nhr-49***	Reduced fecundity, GFP dim	Reduced fertility, worms small and thin, GFP suppressed
***taf-4***	Delayed egg production, medium GFP induction	Delayed development, sterile, no GFP induction
***tbx-2***	Vector-like	Reduced fertility, weak/medium GFP suppression
***Y62E10A.17***	Vector-like	Delayed development, delayed egg production

* Vector control = pL4440-containing HT115 bacteria cultured on RNAi plates (see Materials & Methods for details).

** daf-2 is not a transcription factor. It was included in the screen as a blind control.

*** Both elt-3 and nhr-105 showed an unusual suppression of background GFP fluorescence in head hypodermis. Background staining in remaining hypodermal cells appeared normal.

**Figure 2:**
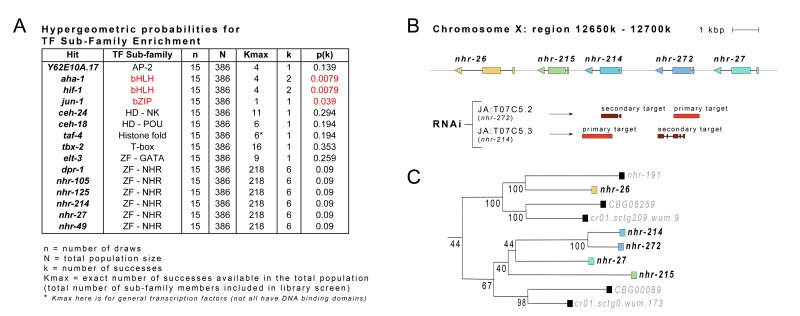
Transcription Factor Sub-Families are Enriched Within Set of Identified RNAi Clones (**A**) Hypergeometric probability analysis was performed on the 15 transcription factors identified in our phenotypic screen. RNAi targeting genes encoding transcription factors belonging to the basic helix-loop-helix (HIF-1, AHA-1) and basic leucine zipper domain (JUN-1) sub-families occurred more frequently than expected by chance. (**B**) Locus surrounding *nhr-27* and *nhr-214* on the X chromosome of *C. elegans* (Wormbase, Version: WS236). Secondary RNAi targets are sites where RNAi clones are predicted to cross-react with unintended gene loci (JA: refers to the Julie Ahringer RNAi library clones used in this study). (**C**) Phylogenetic relationship between *nhr-27, nhr-214* and other nuclear hormone receptors. (Tree was constructed by www.treefam.org). Numbers indicate bootstrap values (percentage of times branch pattern was maintained).

Among the set of identified clones, probability analysis using a hypergeometric distribution revealed that RNAi targeting genes encoding transcription factors belonging to the basic helix-loop-helix (HIF-1, AHA-1) and basic leucine zipper domain (JUN-1) sub-families occurred more frequently than expected by chance (*p = 0.0079* and *p = 0.039*, respectively; Figure 2A). RNAi clones targeting six nuclear hormone receptor genes were also retrieved: *dpr-1, nhr-27, nhr-49, nhr-105, nhr-125* and *nhr-214*. Given the nuclear hormone receptor (NHR) bias in our transcription factors sub-library, this represented neither an enrichment nor de-enrichment of these genes (*p = 0.09*). Of particular note, phylogenetic analyses revealed that two of these genes, *nhr-27* and *nhr-214*, encode closely related NHR homologs (Figure 2B,C). The most recent ancestor of *nhr-214* is *nhr-272* but the RNAi targeting this gene was not retrieved in our screen. The six additional RNAi clones that we identified targeted genes encoding two homeodomain-containing proteins (CEH-18 and CEH-24), the GATA-recognizing zinc-finger protein ELT-3, the T-box containing transcription factor TBX-2, TAF-4 which is a component of the TFIID complex that forms part of the general RNA Pol II transcriptional machinery, and finally, an unnamed transcription factor (Y62E10A.17) belonging to the activating protein 2 (AP-2) family of transcription factors. We have called this gene *udi-1* for ‘Unruly Development with *isp-1*’ in reference to the opposite phenotypes observed in *isp-1(qm130)* Po and F_1_ generation animals when fed RNAi against this gene (compare Table I with Supplementary Table I).

**Figure 3:**
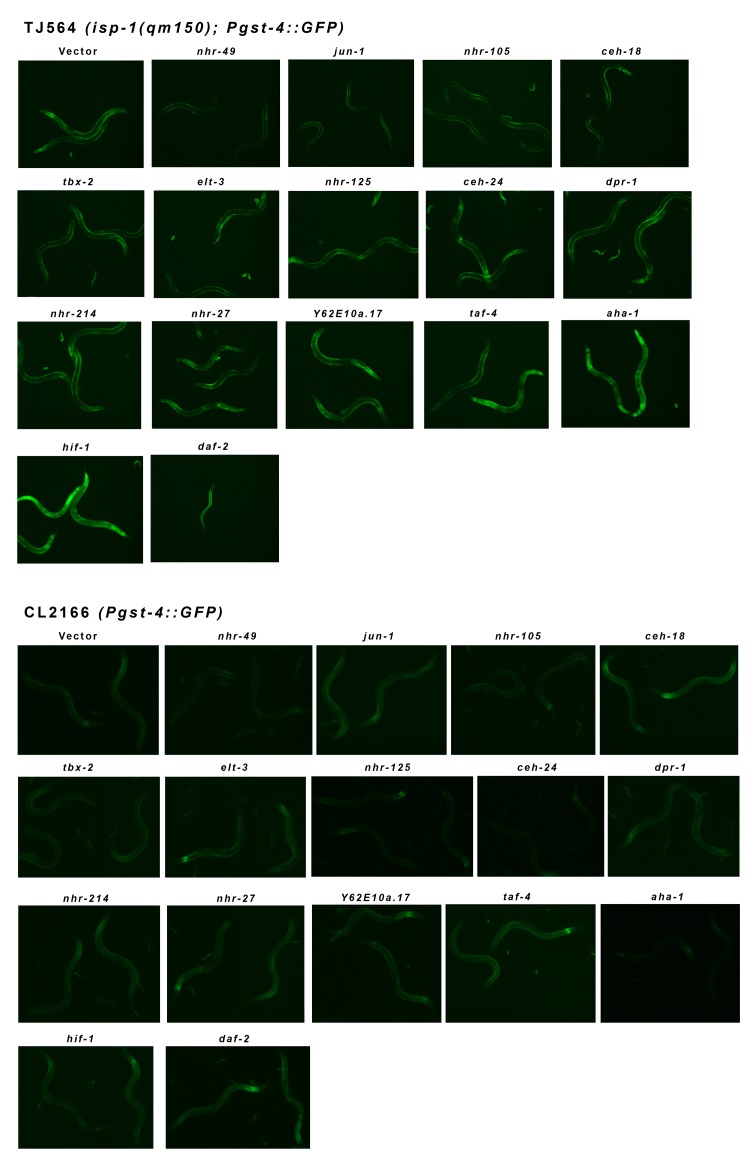
*Pgst-4::GFP* Reporter Expression in isp-1 Mit Mutants is Modulated by Multiple Transcription Factors *isp-1(qm150)* Mit mutants constitutively activate the *Pgst-4::GFP* reporter from the L4 stage to mid-adulthood. Shown are the effects of different transcription factor feeding RNAi treatments on the expression of GFP in one-day adult *isp-1(qm150); Pgst-4::GFP* worms versus control one-day old adult *Pgst-4::GFP* worms. All worms were placed on RNAi lawns as one day arrested L1 and consequently daf-2 RNAi resulted in *isp-1(qm150); Pgst-4::GFP* dauer arrest (compare Supplementary Figure S2). The *Pgst-4::GFP* reporter is constitutively activated in dauer worms (not shown).

Figure 3 shows the effect each of the fifteen RNAi clones had on the expression of the *Pgst-4::GFP* transgene in the *isp-1(qm150)*and control backgrounds. RNAi targeting *hif-1* markedly and robustly induced the *Pgst-4::GFP* reporter in *isp-1* mutants. Knockdown of *aha-1*, which encodes the heterodimeric partner of HIF-1, also induced *Pgst-4::GFP* in these animals, but less strongly than *hif-1*. Loss of *ceh-24, dpr-1, nhr-27* and *nhr-125* all resulted in weak GFP reporter induction in *isp-1* mutants while *elt-3, nhr-49, nhr-105, taf-4* and *tbx-2* all suppressed GFP induction in *isp-1* mutants below background levels. In control animals, a weak induction of GFP was observed when animals were exposed to RNAi targeting *hif-1* while *nhr-49* RNAi resulted in a slight reduction compared to background levels. Finally, *elt-3* and *nhr-105* reproducibly and specifically decreased the background staining of GFP in hypodermal cells of the head only (Figure 3 and Supplementary Figure S1 A).

During our screening procedure, we made an unexpected finding: the method by which Po animals were prepared altered the final effect of some RNAi clones (Supplementary Figure S1). This effect was most evident in *isp-1(qm150); Pgst-4::GFP* animals fed *daf-2* RNAi. In *C. elegans, daf-2* encodes the sole insulin/IGF-like receptor tyrosine kinase and RNAi targeting this gene was originally included in our screen as a control. When *isp-1(qm150); Pgst-4::GFP* animals were placed on *daf-2* RNAi as 24 hour-arrested L1 larvae, a synthetic dauer arrest phenotype was observed (Table I, Figure 3). If animals were instead placed on the lawns as eggs, so that larvae hatched directly onto their RNAi food source, these animals developed into fertile adults (Supplementary Figure S1 B). A previous study reported *daf-2(e1370); isp-1(qm130)* double mutants form fertile adults with a lifespan extension roughly additive of the two single mutants' [10]. That study also reported that *isp-1(qm150)* mutants remain long-lived, even in the presence of a *daf-16* loss of function mutation, which is epistatic to *daf-2*. These findings have been used to argue against a role for insulin/IGF-1 like signaling in Mit mutant longevity. Our current findings now show that early during larval development, the Daf and Mit longevity pathways do, in fact, interact.

### Seven transcription factors modulate *isp-1(qm150)* life extension

**Figure 4:**
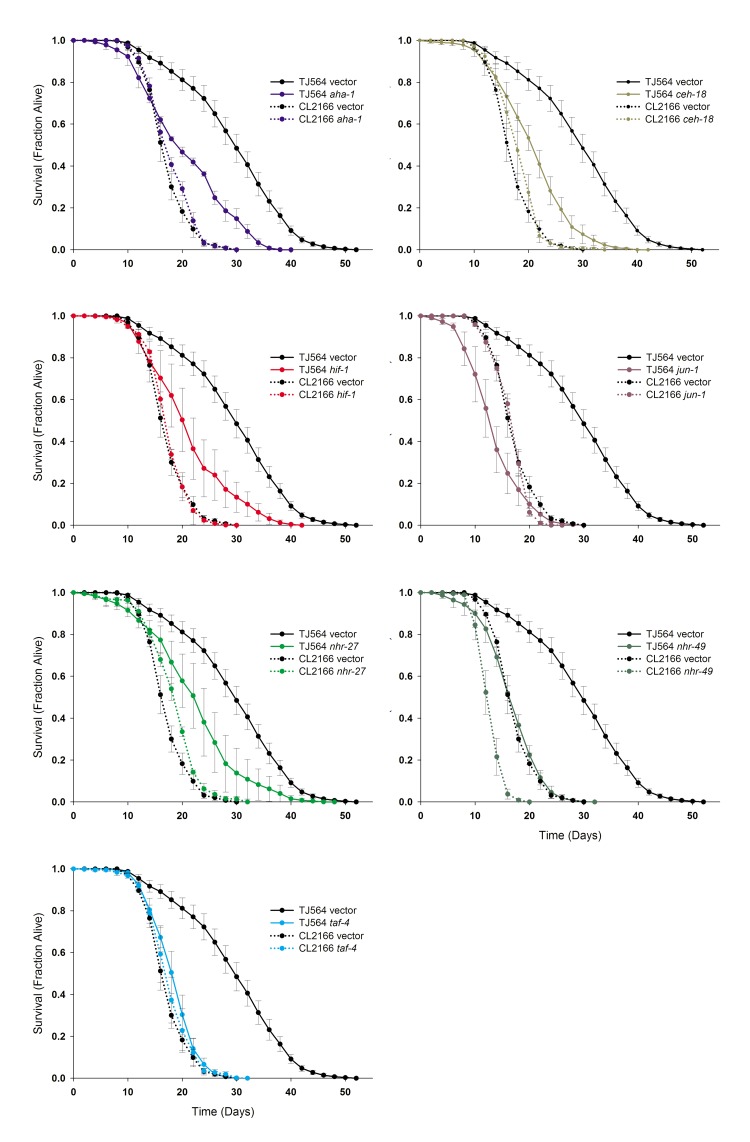
*isp-1* Life Extension is Under the Control of Multiple Transcription Factors Seven transcription factors significantly shortened the lifespan of *isp-1; Pgst-4::GFP* Mit mutants relative to *Pgst-4::GFP* worms when tested across multiple, independent replicates. (**A-G**) Survival curves are presented as average of multiple replicates (≥3 replicates per condition, ±S.E., N=60 worms/replicate) for each strain on each of the seven RNAi clones (strain and RNAi clone are marked) as well as vector control. Complete lifespan data are provided in Supplementary File 3 and summarized in Table II. RNAi-mediated knock-down of *nhr-49* significantly shortened the lifespan of both strains. *daf-2* RNAi was included as a positive control.

Having identified a set of transcription factors that alter various aspects of the Mit phenotype in *isp-1* mutants, we next sought to determine if any of these proteins also play a role in the extended lifespan of these animals. We performed lifespan analyses on over six thousand *isp-1(qm150); Pgst-4::GFP* and control *Pgst-4::GFP* animals that encompassed multiple replicates of each strain cultured on the fifteen different transcription factor RNAi clones. Replicate experiments (≥3) were performed by multiple investigators (Figure 4, and Supplementary Figures S3-S5) so that only the most robust effects were likely to be observed. We identified seven transcription factors that are required for *isp-1* life extension. Knockdown of *aha-1, ceh-18, hif-1, jun-1, nhr-27, nhr-49* and *taf-4* all resulted in a greater reduction of *isp-1* lifespan than wild-type (descriptive statistics and significance values are provided in Table II and Supplementary File 3). Of these RNAi treatments, *jun-1* and *taf-4* knockdown completely abolished the increased lifespan of *isp-1(qm150);Pgst-4::GFP* mutants.

**Table II:** Lifespan Data for All Strains Strain: CL2166 *(gst-4::GFP)*

Combined Survival Statistics (*n_TOTAL_* = 3135)					Stratified* Cox Hazard Model Estimates			
RNAi	Number of Worms (censored)	Mean Expected Lifespan (Days)	Standard Error	Log Rank (vs.Vector)**	Hazard Ratio (RNAi/Vector)	95% C.I. (Lower)	95% C.I. (Upper)	*p* Value***
***aha-1***	180 *(27)*	17.68	0.31	4.9E-02	0.777	0.619	0.975	***0.03***
***ceh-18***	181 *(101)*	18.37	0.48	3.4E-02	0.64	0.486	0.841	***0.00***
***ceh-24***	190 *(50)*	18.94	0.32	2.0E-03	0.639	0.506	0.806	***<0.001***
***daf-2***	180 *(3)*	29.03	0.49	***5.2E-63***	0.113	0.088	0.146	***<0.001***
***dpr-1***	181 *(30)*	19.26	0.34	***1.6E-05***	0.574	0.457	0.722	***<0.001***
***elt-3***	182 *(32)*	18.32	0.37	8.9E-03	0.634	0.504	0.797	***<0.001***
***hif-1***	180 *(38)*	17.93	0.29	5.1E-01	0.938	0.745	1.181	0.59
***jun-B***	180 *(51)*	17.27	0.26	6.8E-01	1.052	0.83	1.333	0.67
***nhr-105***	180 *(28)*	17.38	0.34	6.7E-01	0.848	0.674	1.066	0.16
***nhr-125***	180 *(24)*	19.11	0.33	***1.5E-04***	0.591	0.471	0.742	***<0.001***
***nhr-214***	180 *(16)*	18.99	0.29	***2.8E-04***	0.686	0.549	0.857	***<0.001***
***nhr-27***	180 *(38)*	18.83	0.36	***2.3E-04***	0.61	0.484	0.769	***<0.001***
***nhr-49***	180 *(10)*	13.48	0.16	***6.4E-38***	4.738	3.768	5.958	***<0.001***
***taf-4***	181 *(13)*	18.25	0.31	6.8E-03	0.746	0.597	0.932	***0.01***
***tbx-2***	180 *(52)*	17.37	0.34	9.3E-01	0.923	0.728	1.17	0.51
***Y62E10A.17***	180 *(16)*	18.09	0.29	9.8E-02	0.848	0.679	1.06	0.15
**Vector**	241 *(41)*	17.71	0.25	-	1	-	-	-

**Table:** Strain: TJ564 *[isp-1(qm150); gst-4::GFP]*

Combined Survival Statistics (*n_TOTAL_* = 3256)					Stratified* Cox Hazard Model Estimates			
RNAi	Number of Worms (censored)	Mean Expected Lifespan (Days)	Standard Error	Log Rank (vs.Vector)**	Hazard Ratio (RNAi/Vector)	95% C.I. (Lower)	95% C.I. (Upper)	*p* Value***
***aha-1***	189 *(29)*	21.24	0.72	***5.7E-11***	1.634	1.312	2.035	***<0.001***
***ceh-18***	185 *(10)*	21.81	0.49	***5.5E-31***	2.303	1.853	2.862	***<0.001***
***ceh-24***	180 *(9)*	27.47	0.61	***5.5E-06***	0.983	0.792	1.22	0.88
***daf-2***	152 *(47)*	40.15	1.25	***2.4E-21***	0.216	0.161	0.289	***<0.001***
***dpr-1***	184 *(23)*	29.53	0.57	2.5E-02	0.849	0.682	1.056	0.14
***elt-3***	185 *(19)*	29.75	0.58	2.2E-02	0.84	0.677	1.043	0.12
***hif-1***	180 *(9)*	22.25	0.55	***3.3E-23***	2.35	1.894	2.916	***<0.001***
***jun-B***	183 *(24)*	14.59	0.38	***4.5E-87***	10.205	8.101	12.855	***<0.001***
***nhr-105***	180 *(12)*	27.75	0.64	***3.1E-04***	0.936	0.753	1.164	0.55
***nhr-125***	180 *(18)*	28.31	0.67	5.4E-03	0.95	0.764	1.182	0.65
***nhr-214***	183 *(35)*	27.73	0.65	3.0E-03	1.123	0.9	1.402	0.30
***nhr-27***	182 *(18)*	23.13	0.66	***1.3E-13***	1.752	1.409	2.178	***<0.001***
***nhr-49***	195 *(2)*	17.76	0.36	***3.7E-69***	5.027	4.045	6.246	***<0.001***
***taf-4***	180 *(41)*	19.24	0.33	***7.3E-53***	4.356	3.446	5.508	***<0.001***
***tbx-2***	180 *(20)*	28.74	0.66	1.8E-02	0.912	0.732	1.136	0.41
***Y62E10A.17***	180 *(11)*	28.66	0.57	***3.8E-06***	1.151	0.929	1.427	0.20
**Vector**	358 *(21)*	30.21	0.50	-	1	-	-	-

**Table:** Strain: MQ770 *[tpk-1(qm162), III]*

Combined Survival Statistics (*n_TOTAL_* = 1085)					Stratified* Cox Hazard Model Estimates			
RNAi	Number of Worms (censored)	Mean Expected Lifespan (Days)	Standard Error	Log Rank (vs.Vector)**	Hazard Ratio (RNAi/Vector)	95% C.I. (Lower)	95% C.I. (Upper)	*p* Value***
***aha-1***	120 *(13)*	28.86	0.76	***4.2E-04***	1.351	1.037	1.76	***0.026***
***ceh-18***	120 *(15)*	30.03	0.76	***2.4E-04***	1.353	1.037	1.763	***0.026***
***hif-1***	121 *(11)*	29.26	0.68	***1.3E-04***	1.458	1.121	1.895	***0.005***
***jun-1***	120 *(6)*	20.95	0.48	***4.7E-41***	5.684	4.294	7.523	***<0.001***
***nhr-105***	120 *(4)*	27.94	0.71	***4.8E-09***	1.946	1.498	2.527	***<0.001***
***nhr-27***	121 *(5)*	33.17	0.74	8.3E-01	0.885	0.683	1.147	0.356
***taf-4***	120 *(2)*	21.36	0.44	***4.5E-41***	5.739	4.342	7.585	***<0.001***
**Vector**	243 *(16)*	32.15	0.56	-	1	-	-	-

**Table:** Strain: MQ130 *[clk-1(qm30)]*

Combined Survival Statistics (*n_TOTAL_* = 540)					Stratified* Cox Hazard Model Estimates			
RNAi	Number of Worms *(censored)*	Mean Expected Lifespan (Days)	Standard Error	Log Rank (vs. Vector)**	Hazard Ratio (RNAi/Vector)	95% C.I. (Lower)	95% C.I. (Upper)	*p* Value***
***aha-1***	60 *(1)*	22.69	1.49	4.3E-01	1.223	0.85	1.759	0.277
***ceh-18***	60 *(2)*	23.54	1.17	5.0E-01	1.19	0.824	1.72	0.353
***hif-1***	60 *(2)*	20.36	1.12	***1.7E-03***	1.834	1.268	2.653	***0.001***
***jun-1***	60 *(2)*	26.17	0.96	6.2E-01	1.128	0.779	1.633	0.525
***nhr-105***	60 *(1)*	22.55	1.55	3.6E-01	1.213	0.845	1.742	0.294
***nhr-27***	60 *(1)*	24.57	1.15	8.3E-01	1.048	0.727	1.51	0.803
***taf-4***	60 *(2)*	19.01	0.73	***7.6E-04***	1.954	1.339	2.85	***<0.001***
**Vector**	120 *(2)*	24.06	1.04	-	1	-	-	-

**Table:** Strain: SLR0055*[clk-1(qm30)Pgst-4::GFP]*

Combined Survival Statistics (*n_TOTAL_* = 545)					Stratified* Cox Hazard Model Estimates			
RNAi	Number of Worms *(censored)*	Mean Expected Lifespan (Days)	Standard Error	Log Rank (vs.Vector)**	Hazard Ratio (RNAi/Vector)	95% C.I. (Lower)	95% C.I. (Upper)	*p* Value***
***aha-1***	59 *(10)*	22.43	0.76	5.6E-01	1.043	0.716	1.521	0.825
***ceh-18***	61 *(7)*	30.44	1.44	***2.8E-09***	0.258	0.17	0.391	***<0.001***
***hif-1***	61 *(5)*	27.20	0.99	***1.8E-03***	0.535	0.369	0.775	***<0.001***
***jun-1***	60 *(5)*	23.05	0.78	6.4E-01	0.904	0.627	1.303	0.588
***nhr-105***	61 *(8)*	27.03	0.95	6.1E-03	0.557	0.384	0.809	***0.002***
***nhr-27***	60 *(19)*	25.45	0.96	2.0E-02	0.656	0.441	0.977	***0.038***
***taf-4***	60 *(4)*	19.55	0.44	***2.4E-04***	1.805	1.24	2.627	***0.002***
**Vector**	122 *(6)*	23.18	0.56	-	1	-	-	-

* **Stratified by experiment,**

** **Bold italic indicates significant after controlling for multiple comparisons,**

*** **Bold italic indicates significant (*p*<0.05).**

In particular, *jun-1* RNAi pathologically shortened lifespan. Both RNAi treatments were without effect on *Pgst-4::GFP*control animals. RNAi knockdown of *nhr-49* significantly reduced the lifespan of both strains while, as expected, *daf-2* knockdown significantly increased the lifespan of both strains (in *isp-1* animals that did not experience dauer arrest). The lifespan increase by *daf-2* was additive with *isp-1*. Interestingly, RNAi targeting *dpr-1, nhr-27, nhr-125* and *nhr-214* all marginally (~6%), but significantly *(p<0.0003)*, increased the lifespan of *Pgst-4::GFP* animals (Table II and Supplementary Figure S5).

### Mit mutant life extension is mediated by a general set of transcription factors

**Figure 5:**
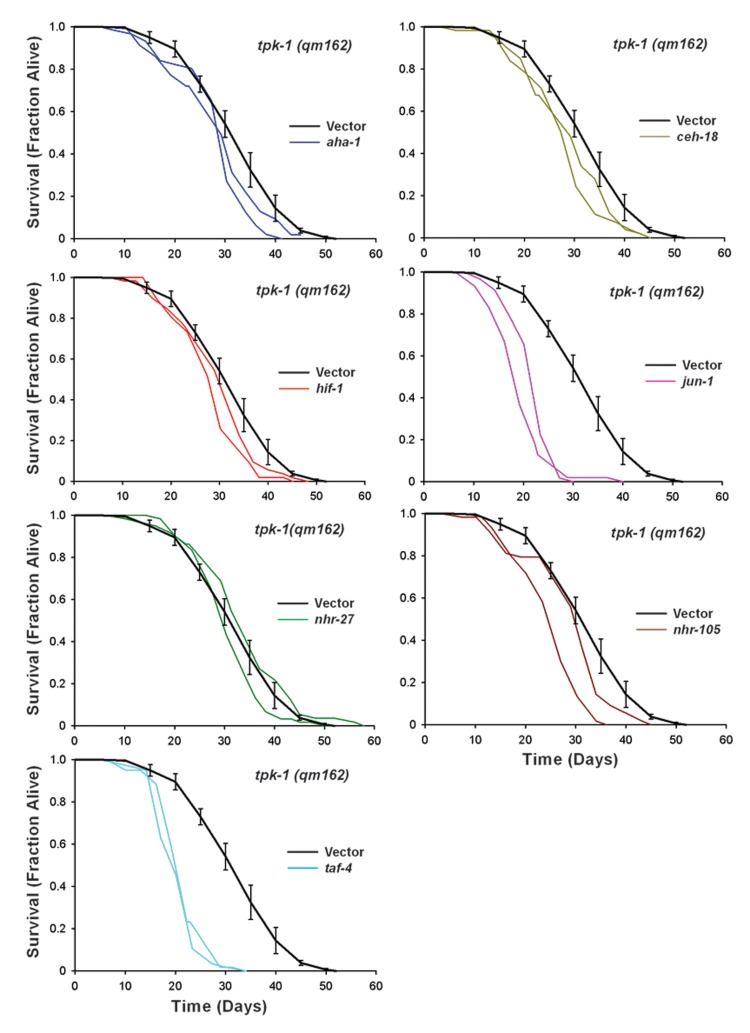
Six Transcription Factors Required for *isp-1(qm150)* Life Extension are also Required for *tpk-1(qm162)* Life Extension Replicate survival plots for *tpk-1(qm162)* worms cultured on RNAi targeting transcription factors that differentially shortened the lifespan of *isp-1* mutants. Knock-down of six transcription factors, *taf-4, jun-1, hif-1, aha-1, ceh-18*, as well as *nhr-105*, each significantly reduced *tpk-1* lifespan. *nhr-27* RNAi was without effect (N=60 worms/trace). Vector-only RNAi is included in each plot as the relevant control and the single curve represents combined data from four independent assays (error bars: +/−SEM, N=262 worms total). Complete lifespan data are provided in Supplementary File 3 and summarized in Table II.

**Figure 6:**
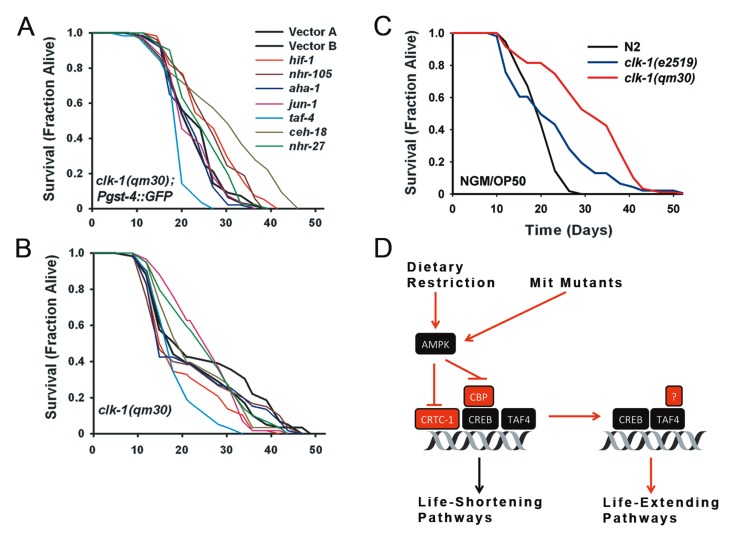
TAF-4 and HIF-1 are Required for *clk-1(qm30)* Life Extension **(a)** Survival curves for *clk-1(qm30); Pgst-4::GFP* transgenic worms cultured on RNAi targeting each of seven transcription factors required for *isp-1(qm150)* life extension. For this panel, as well (b) and (c), a summary of lifespan data and significance testing is provided in Supplementary File 3 and Table II. **(b)** In the absence of the *Pgst-4::GFP* reporter, *clk-1(qm30)* remains surprisingly recalcitrant to any life-shortening effect of the seven RNAi clones. Only *taf-4* and *hif-1* RNAi significantly shorten lifespan. **(c)** Survival analysis of *clk-1(qm30), clk-1(e2519)* and N2 wild-type animals cultured on standard NGM/OP50 plates. Mean lifespan of each strain is 29.5, 21.3, 18.8 days, respectively. Both *qm30* and *e2519* are significantly longer-lived than N2 (*p=0.049* and *1.3E-09*, respectively. (N=60 worms/trace, for all panels). (D) Model depicting role of TAF-4 in Mit mutant life extension. TAF-4 is a component of the general transcription machinery leading to recruitment of RNA Pol II to basal promoters. CREB contains binding sites for TAF-4, CRTC-1 and CBP. AMPK activation inhibits the interaction of CBP and CRTC-1 with CREB. Loss of TAF-4 blocks Mit mutant life extension, so too does removal of AMPK.

We next tested the generality of *aha-1, ceh-18, hif-1, jun-1, nhr-27*, and*taf-4* in the life extension of two other Mit mutants: *tpk-1(qm162)* and*clk-1(qm30)*. We included RNAi targeting *nhr-105* in this analysisalso, since knock-down of this transcription factor caused a marginally significant reduction in *isp-1* lifespan (Table II). Survival curves for *tpk-1(qm162)*and *clk-1(qm30)* are presented in Figures 5 and 6, respectively. Summary statistics and significance testing are described in Table II. All RNAi clones except *nhr-27* significantly reduced the lifespan of *tpk-1* mutants. As was observed for *isp-1(qm150)*, RNAi targeting *jun-1* and *taf-4* both caused potent life shortening in *tpk-1(qm162)* mutants.

The situation for *clk-1(qm30)* mutants was considerably different. In *clk-1(qm30); Pgst-4::GFP* animals, only knockdown of *taf-4* significantly shortened lifespan (Figure 6A, Table II). Unexpectedly, knockdown of *ceh-18, hif-1, nhr-105* and *nhr-27* all resulted in a significant extension of lifespan. For *nhr-27*, this effect was similar to that seen for control *Pgst-4::GFP* animals (Figure 4). When we repeated this experiment using *clk-1(qm30)* mutants without the *Pgst-4::GFP* reporter in the background, again *taf-4* RNAi significantly shortened lifespan, but so too did RNAi targeting *hif-1* (Figure 6B, Table II); none of the other RNAi clones altered lifespan. Surprised by the shape of the survival curves for the *clk-1(qm30)* strain (which is suggestive of two sub-populations [25]), and by the overall weakness of the life extension observed in both strains when cultured on vector-only RNAi, we decided to test the lifespan of the *clk-1(qm30)* strain when grown on standard NGM/OP50 plates. Under these conditions robust extension of life was observed (Figure 6C), as reported previously [11]. Given that *clk-1(qm30)* acquires its ubiquinone from its bacterial food source [26], the most likely explanation for these unexpected results is that the HT115 bacteria used for feeding RNAi purposes more effectively rescues the ubiquinone deficit of *clk-1(qm30)* animals than does OP50 bacteria, in effect, making them only weak Mit mutants. Consistent with this notion, we found that the loss of function *qm30* allele grown on HT115 had survival kinetics more akin to the hypomorphic *e2519* allele cultured on OP50 (Figure 6C). Based on our current results, we conclude that at least two transcription factors, TAF-4 and HIF-1, appear to be generally required for Mit mutant life extension. Four other transcription factors, AHA-1, CEH-18, NHR-105 and JUN-1, are also likely to be important mediators of lifespan in this class of long-lived *C. elegans* mutants.

## DISCUSSION

Since the discovery of the first Mit mutant almost twenty years ago [11], significant effort has been expended in trying to determine why this group of worms are long-lived. These animals met early resistance as a relevant model to study aging since, at the time, it was almost unfathomable that a mutant defined by mitochondrial ETC dysfunction could also be long-lived. With time it became clear that life extension in the face of ETC disruption was not peculiar to *C. elegans* [4, 6], that manifestation of the Mit phenotype is a threshold effect phenomenon [3], and that multiple processes are activated in these animals to mitigate the detrimental effects of ETC loss with the net consequence being an extension of lifespan [3, 8, 14-18]. Our present studies now extend considerably our understanding of the transcription factor network underlying appearance of the Mit phenotype. Through a focused RNAi screen, we have uncovered a trove of DNA-binding proteins that affect various aspects of the Mit phenotype in *isp-1(qm150)* mutants. We have identified fifteen transcription factors in total, seven of which are required for *isp-1* life-extension, and two of these, namely TAF-4 and HIF-1, are also required to extend the lifespan of two other Mit mutants, *tpk-1(qm162)* and *clk-1(qm30)*.

HIF-1α is a conserved transcription factor required in many organisms for survival under low oxygen conditions. In worms, HIF-1α (HIF-1) controls the expression of over 60 genes following acute hypoxia [27]. Under normoxic conditions, HIF-1 is rapidly degraded, but in several RNAi-induced Mit mutants, as well as in two genetically-defined Mit mutants – *isp-1(qm150)* and *clk-1(qm30)* – HIF-1 is constitutively active [16]. Inhibition of HIF-1 by RNAi in *isp-1(qm150)* and *clk-1(qm30)* Mit mutants negated their life extension. Additionally, stabilization of HIF-1 in wild-type animals, by inhibition of either VHL-1 or EGL-9 (which encode the E3 ubiquitin ligase and the prolyl hydroxylase required for HIF-1 degradation, respectively), was sufficient to increase the lifespan of wild-type worms but did not further increase the lifespan of Mit mutants. In this study, we independently identified HIF-1 to be required for the extended lifespan of three different genetic Mit mutants: *isp-1(qm150), tpk-1(qm162)* and *clk-1(qm30)*. We also examined oxidative stress levels in control and *isp-1* mutant worms by utilizing the *Pgst-4::GFP* reporter that was integrated into these strains. The *isp-1(qm150)* mutants had a high basal level of GFP expression compared to control worms, suggesting that *isp-1* mutants are in a state of heightened oxidative stress. RNAi knockdown of HIF-1 had only a mild effect on GFP expression in control worms, while in *isp-1* mutants, knockdown of HIF-1 resulted in a strong GFP signal. This suggests that in *isp-1(qm150)* Mit mutants, functional HIF-1 may be part of a compensatory response that mitigates some of the oxidative stress created by mitochondrial dysfunction. This finding is consistent with recent suggestions that Mit mutant life extension requires ROS signaling [16].

Some of the other transcription factors that we found to be essential for lifespan extension of *isp-1(qm150)* mutants are AHA-1, CEH-18, JUN-1, NHR-27 and NHR-49. AHA-1 is the worm ortholog of HIF-1β. Under hypoxic conditions, when HIF-1α is stabilized, both proteins form a complex that binds DNA and leads to transcription of genes under the control of the HIF Response Element (HRE) [27]. A previous study revealed that AHA-1 is required for the lifespan extension of *isp-1(qm150)* and *clk-1(qm30)* mutants [16]. Our study corroborates that finding; we show that AHA-1 is required for the lifespan extension of *isp-1(qm150)* mutants, as well as *tpk-1(qm162)* mutants. CEH-18 is another transcription factor that we found to be required for the life extension of both *isp-1(qm150)* and *tpk-1(qm162)* Mit mutants. This protein is known to have a role in oocyte development, where it acts to prevent oocyte maturation and ovulation in the absence of sperm. [28]. In our experiments, *ceh-18* RNAi reduced fertility and affected development of both wild-type and *isp-1* mutants (Table I) to differing extents. Previously, Hamilton and colleagues showed that *ceh-18* RNAi caused a significant extension in mean lifespan of wild-type worms (from 12.3 to 14.5 days) [9]. We also noted a slight increase of lifespan in wild-type worms treated with *ceh-18* RNAi (Table II), but this effect was not significant after controlling for multiple comparisons.

We found JUN-1 to also be required for the life extension of *isp-1(qm150)* and *tpk-1(qm162)* Mit mutants. JUN-1 is the worm ortholog of the human transcription factor JunB. In mammalian cells, JunB is important for cell cycle regulation and for differentiation in response to growth factors such as transforming growth factor beta-1 (TGFβ1) [29]. In *C.elegans*, JUN-1 was recently shown to mediate life extension in response to intermittent fasting [30].

NHR-27 and NHR-49 are two more transcription factors identified by our screen. Very little is known about the role of NHR-27 in worms. Its closest human homolog is NR1H4, which is a nuclear bile acid receptor also known as Farnesoid X receptor (FXR). NR1H4 is activated by intracellular bile acids and, in response, regulates expression of a number of bile acid responsive genes, mainly in liver and intestinal cells [31]. Recent studies have uncovered a bile acid-like biosynthetic pathway in worms that governs longevity through the gonad [32]. With regards to NHR-49, this protein is known to regulate fatty acid metabolism in *C.elegans* in response to changes in nutrient status [33]. In several RNAi-induced Mit mutants, NHR-49 was shown to mediate a starvation-like response that included induction of genes required for β-oxidation, the glyoxylate cycle, gluconeogenesis and glycolysis [34]. Surprisingly, NHR-49 was not required for the life extension of at least four of these RNAi-induced Mit mutants (*nuo-1, ucr-1, cyc-1* and *cco-3)*, but it is worth noting that the specific mutant allele employed in those studies affected only three of four NHR-49 splice variants. The effect of NHR-49 loss on genetically-defined Mit mutants had not been tested until now.

The requirement for TAF-4 in Mit mutant longevity has not been previously reported. TAF-4 is a ubiquitously expressed component of the general transcription factor TFIID that participates in formation of the pre-initiation complex necessary for transcription by RNA polymerase II (pol II) [35]. TFIID is comprised of the TATA-binding protein (TBP) and a group of 14 subunits called TBP-associated factors (TAFs), of which TAF-4 is one. Work in *C. elegans* suggests that TAF-4 is essential for all RNA Pol II transcription in the early embryo [36]. Presumably, this requirement is relaxed after embryonic development since we have now observed that L1 hatchlings exposed to TAF-4 RNAi throughout their remaining life grow normally and show no overt phenotype beyond reduced fertility. Consistent with this finding, mice null for *TAF4* are embryonic lethal, but fibroblasts derived from TAF4 null embryos are viable and grow under normal tissue culture conditions [37]. Wright and colleagues showed that TAF4 plays a significant role in mediating transcription from a TATA-less downstream core promoter element, but in TATA-containing promoters, TAF4 was not essential for transcription [38]. In adult mice, TAF subunits show differential expression in a variety of tissues [39], suggesting that the TAFs may selectively and differentially activate tissue specific transcription. Recent studies also show that TAFs may have specificity toward particular core promoters: Gazit and colleagues showed that the TAFs bind DNA with high affinity and have a weak sequence preference [40]. They also showed that TAF4 is required for core promoter function of not all, but only a subset of genes.

One such subset of genes that require TAF4 as a coactivator are genes that are under the control of the glutamine-rich transcription factor CREB (cyclic AMP response element binding protein) [41]. CREB is activated in response to a multitude of extracellular signals including growth factors, hormones and stress signals [42]. CREB and CREB-regulated transcriptional coactivators (CRTCs) control genes involved in a variety of physiological functions including energy homeostasis, carcinogenesis, ER stress, immune function and neurogenesis. Mengus and colleagues showed that induction of CREB transcriptional activity is strongly diminished in *taf4^−/−^* cells [37]. The role of TAF4 as a coactivator for CREB is important in the context of our findings. CREB is known to be induced by mitochondrial dysfunction [43]. Recently, CREB has been shown to mediate lifespan extension in *C. elegans* downstream of AMPK (5' AMP mediated protein kinase), which is a master regulator of cellular energy balance [19] and essential for the longevity of *isp-1(qm150)* and *clk-1(qm30)* Mit mutants [18]. CRTC-1 binds to and activates CREB, leading to transcription of target genes downstream of CREs (cyclic AMP response elements) [44]. Mair and colleagues showed that AMPK phosphorylates CRTC-1, thereby preventing CRTC-1 translocation to the nucleus [19]. They proposed that this is how AMPK blocks activation of CREB to extend lifespan. Paradoxically, our results show that TAF-4, which activates CREB, is required for extended lifespan in Mit mutants. Considering the diversity of functions of the various CREB target genes, we suggest that CREB activation of certain target genes can lead to reduced lifespan, while CREB activation of alternate target genes can lead to increased lifespan. The fact that in the Mair et al. study CREB knockdown resulted in increased lifespan, while complete loss of CREB did not, supports the idea that CREB may be playing a dual role in modulating longevity. The Mit mutants may be mediating their life extension through pathways that lead to activation of particular CREB-target genes that are different from those controlled by CRTC-1. Since TAF-4 is required for this activation, knockdown of TAF-4 prevents Mit mutant life extension (Figure 6D). Apart from CRTC, CREB has another signal-responsive coactivator, CREB Binding Protein (CBP), which has its own binding site on CREB. A recent study suggests that while CRTC can rescue the activation of some CREB target genes in the absence of CBP, there are some CREB target genes that require CBP for activation [45]. CBP and CRTC therefore have some degree of selectivity towards which CREB target genes they activate in response to various signals. What role CBP plays along with TAF-4 in the lifespan modulation of Mit mutants remains an interesting area of further study.

In summary, using a targeted RNAi screening approach, we have identified multiple transcription factors whose functions are important for the life extension of one or more Mit mutants. These include seven transcription factors essential to *isp-1(qm150)* mutants, six of which are also important in *tpk-1(qm162)* mutants. Previously, HIF-1 was shown to be required for longevity in Mit mutants, JUN-1 in intermittent feeding, NHR-49 genes in nutrient status, and NHR-27 in signals from the germline. The fact that all these genes were identified validates our screening approach and emphasizes that it is not one gene or pathway responsible for life extension in Mit mutants, but a network of genes that must be specifically modulated. Nowhere is this more evident than in the case of CREB, which can act either to extend life or shorten it. TAF-4 is one of CREB's regulators and was identified by our screen as being required for Mit mutant longevity. This role of TAF-4 in lifespan determination is novel and our future studies will explore its interactions with other proteins as well as the downstream genes it regulates. Our findings support a retrograde response model of Mit mutant longevity, whereby reduced mitochondrial function leads to activation of various transcription factors that alter gene expression in order to promote survival and increase longevity.

## MATERIALS AND METHODS

### Nematode Strains and Maintenance

The following *C. elegans* strains were utilized in this study: N2 Bristol (wild-type), CB4876 *[clk-1(e2519)III]*, CL2166 *[dvIs19[pAF15(Pgst-4::GFP::NLS)]III]*, MQ130 *[clk-1(qm30)III]*, MQ770 *[tpk-1*(*qm162)III]*, SLR0054 *[dvIs19[pAF15(Pgst-4::GFP::NLS)]III; isp-1(qm150); dpy-13(e184); skn-1(zu67)]IV/nT1[unc^d^(n754); let](IV;V)]*, SLR0055 [*clk-1(qm30)III; dvIs19[pAF15(Pgst-4::GFP::NLS)]III]*, and TJ564 [*isp-1(qm150)IV; dvIs19[pAF15(Pgst-4::GFP::NLS)]III]*. CL2166 behaves indistinguishably from N2 and contains a stably integrated *Pgst-4::GFP* transcription-al reporter. Use of this transgene to measure endogenous oxidative stress has been described previously [20, 21]. TJ564 was backcrossed once into N2 before screening; CL2166 was backcrossed five times. All strains were maintained on standard NGM agar supplemented with *Escherichia coli* (OP50), as described previously [22].

### Transcription Factor Selection and RNAi Screening

429 RNAi clones targeting 386 transcription factors (TFs), 19 transcriptional co-activators, and 22 other transcription-related genes, were identified in the Ahringer *C.elegans* bacterial feeding-RNAi library through a combination of computational searches and manual curation (Supplementary File 1). RNAi clones were retrieved from the library and re-arrayed into 96 well format (Supplementary File 2). Bacteria were grown at 37°C in LB broth supplemented with 100 μg/ml ampicillin and 5 μg/ml tetracyclin for 18 hours. OD_590 nm_ values were then adjusted to 0.9 and 200 μl of each culture was spotted onto freshly poured 6 cm NGM/agar plates containing 100 μg/ml ampicillin, 5 μg/ml tetracyclin, and 1 mM IPTG to induce dsRNA production. Lawns were allowed to mature overnight at room temperature, then stored at 4°C. For screening purposes, freshly prepared feeding RNAi plates were seeded with arrested (24 hour) CL2166 or TJ564 L1 larvae (Po generation, n ≈ 20). Worms were then monitored over the course of their development until vector-only cultured animals became three-day-old adults. A set of positive and negative control RNAi clones were included with every set of test plates. These were RNAi of known effect and are listed in red in Supplementary File 2. RNAi clones that resulted in a differential phenotype in TJ564 relative to CL2166 were isolated and then passed through several additional rounds of re-testing. A flowchart describing our screening procedure is shown in Figure 1. At the end of the third round of re-testing, a set of twenty-four RNAi clones (including *daf-2*, see below) had been identified. This set was further reduced to fifteen clones after F_1_ generation animals were tested in three additional rounds of screening by an independent investigator (Supplementary Table I). These fifteen RNAi clones were then assayed for their ability to attenuate the life extension of *isp-1, tpk-1* and *clk-1* Mit mutants. SLR0054 animals were initially constructed to expedite screening, however the Dpy phenotype of unbalanced worms made screening difficult and so the two strain strategy described above was eventually employed. All screening was performed blind of RNAi clone identity and each round of Po screening was independently scored by two investigators. A compendium of the effects of all 429 RNAi clones on the Po generation of both CL2166 and TJ564 is provided in Supplementary File 2. Final clone identity was confirmed by sequence analysis.

### Fluorescence Imaging

Initial screening was performed using a PCO SensiCam CCD camera connected to a Zeiss Axioskop. GFP fluorescence images were recorded using an Olympus DP71 CCD camera connected to an Olympus SZX16 fluorescence dissecting microscope. Arrested L1 larvae were placed on RNAi lawns and then photographed when vector-treated worms reached one-day-old adulthood. Where stated, eggs that had been laid over a three hour period onto NGM/OP50 lawns were manually transferred onto RNAi lawns in place of one-day arrested L1 larvae.

### Lifespan Analyses

For lifespan studies, synchronous L1 worms (arrested and staged without the use of bleach), were transferred onto freshly prepared NGM agar plates seeded with the relevant bacterial feeding RNAi (60-80 animals/ 6 cm plate, 20°C). When animals reached the L4/YA molt, they were transferred onto fresh RNAi plates containing 25 μM FuDR. Lifespan analyses were initiated when vector-only-treated animals reached young adulthood. Worms were transferred every fifth day to fresh RNAi plates but typically scored every second or third day. Worms that died of vulval rupture were censored on the day of death. Lifespan data was analyzed in SigmaPlot 11.0 (Systat Software, Inc. San Jose, CA) and significance analysis was assessed using both a log-rank test and a Cox Proportional Hazard Model (stratified by experiment). Descriptive statistics and significance results for all individual lifespans are provided in Supplementary File 3. Data was Bonferroni corrected for multiple comparisons using a pre-correction significance threshold of *p*<0.05.

## SUPPLEMENTARY FIGURES AND TABLES






