# Supplementary material for: TAF-4 is required for the life extension of isp-1, clk-1 and tpk-1 Mit mutants
Source: Aging (Albany NY). 2013 Oct 4;5(10):741–58. doi: 10.18632/aging.100604 (PMC3838777; doi:10.18632/aging.100604)
Supplement: Supplementary file 1 [file aging-05-741-s001.pdf]

SUPPLEMENTARY FIGURES

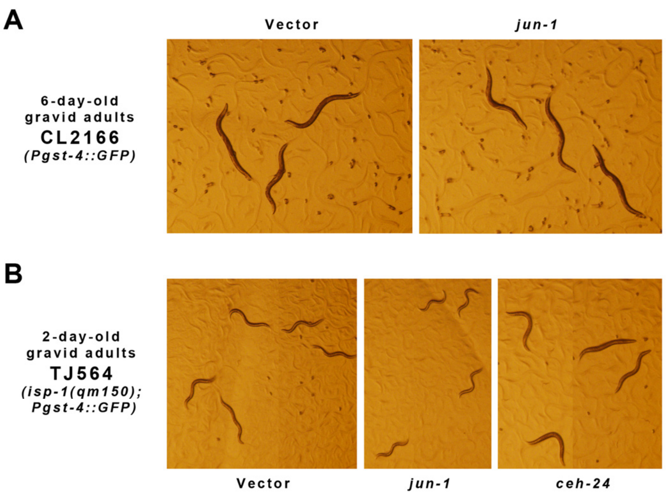

Supplementary Figure S1. Effect of *jun-1* and *ceh-24* Feeding RNAi on *isp-1(qm150)* Adult Size. *isp-1(qm150); Pgst-4::GFP* and control *Pgst-4::GFP* worms populations were cultured on transcription factor feeding RNAi from the time of hatching and then followed over time. **(a)** *Pgst-4::GFP* control worms cultured on *jun-1* RNAi showed no overt phenotypic effects at 6 days of adulthood. **(b)** In contrast, an overt effect of *jun-1* RNAi on *isp-1; Pgst-4::GFP* adult size was evident by day 2 of adulthood. *ceh-24* feeding RNAi, instead, resulted in larger adults and was later found to significantly reduce lifespan (Table II).

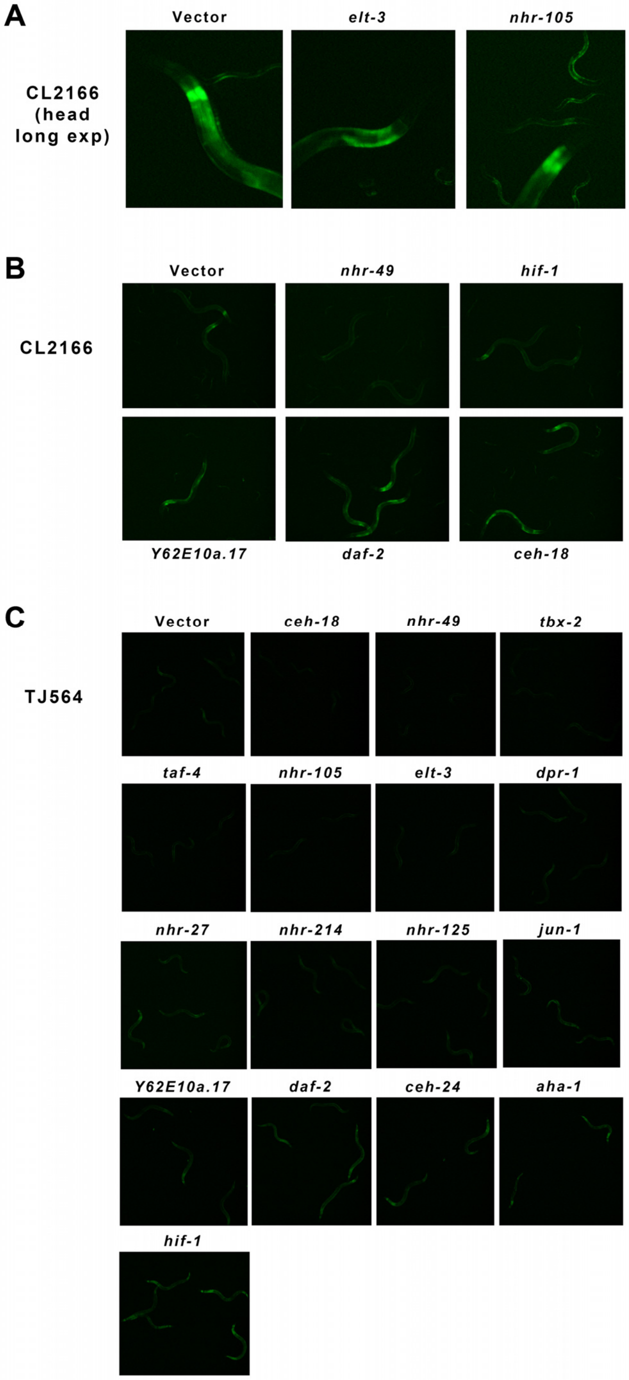

Supplementary Figure S2. Responses to Transcription Factor Feeding RNAi are Influenced by Prior L1 Nutrition. Shown are the effects of transcription factor feeding RNAi on reporter expression in two-day adult *isp-1(qm150); Pgst::GFP* worms versus two-day old adult *Pgst-4::GFP* control worms. Worms were placed on RNAi lawns as eggs and allowed to hatch directly onto food (compare **Figure 3** in which arrested L1 were used). **(a & b)** For *Pgst-4::GFP* worms, *elt-3* and *nhr-105* RNAi overtly blocked GFP expression in head hypodermis. *hif-1* RNAi was without effect, while *udi-1/Y62E10a.17, daf-2* and *ceh-18* RNAi all led to elevated GFP expression. **(c)** For *isp-1;Pgst-4::GFP* worms, *daf-2* RNAi-treated grew into fertile adults, while stronger induction of GFP on *jun-1* RNAi-fed worms was observed.

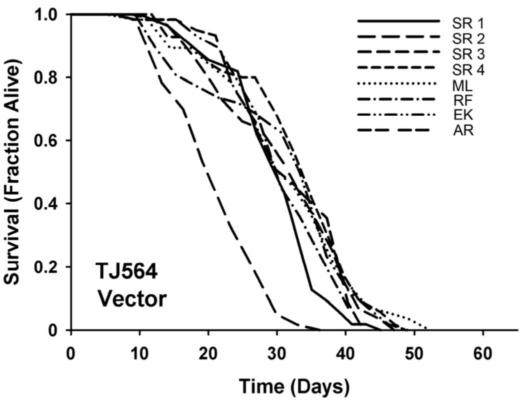

Supplementary Figure S3. Outlier Identification in Survival Analysis of Vector-only treated *isp-1(qm150); gs-4::GFP* worms. Eight independent experiments, separately undertaken by five different investigators (initials), measuring the lifespan of *isp-1(qm150); Pgst-4::GFP* worms cultured on vector-only RNAi-plates illustrate the degree of lifespan variability observed during the course of our studies (*n=60* worms/experiment). Seven of the eight experiments did not differ significantly from each other (*p>0.05,* log rank test). For the eighth experiment, no overt differences in experimental variables could be discerned that distinguished it from the seven other replicate experiments, yet in this particular study worms lived ~10 days shorter, on average. We attribute this difference to chance sampling and consequently chose to leave this experiment in all our analyses (at the risk of losing statistical power). Experiments SR3 and SR4 were part of an unrelated study carried out at the same time and have been published elsewhere. Neither experiment was included in any of the present statistical analyses, but both have been included for comparison in the displays of Figure 4.

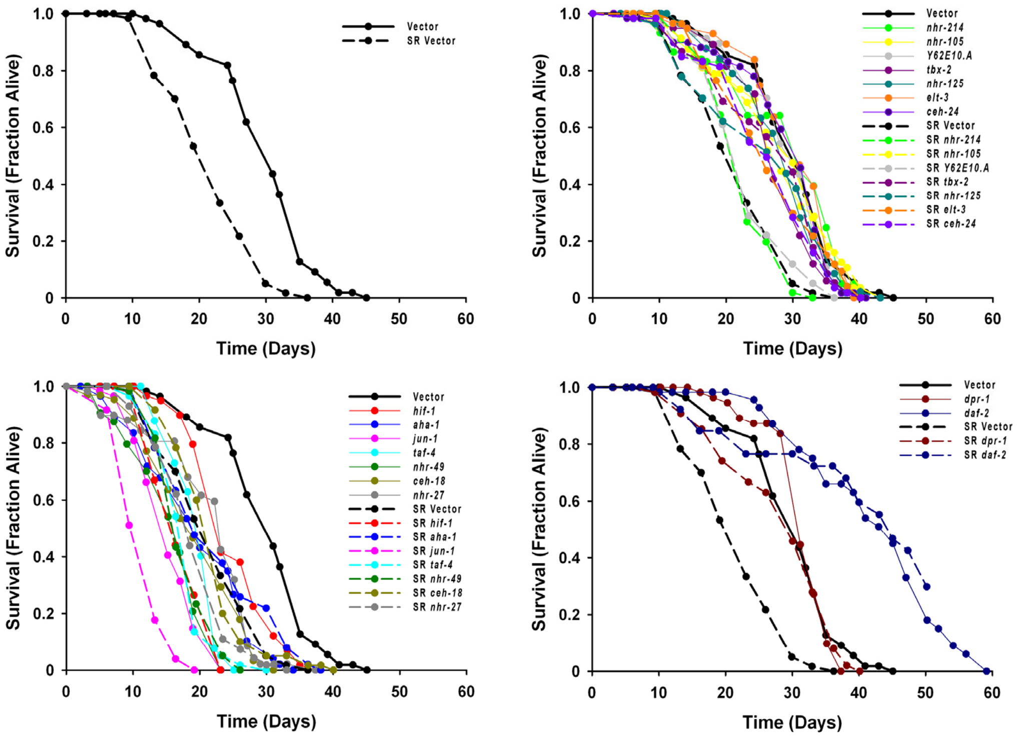

Supplementary Figure S4. *isp-1(qm150); Pgst-4::GFP* Lifespan Studies for all 15 Transcription Factor RNAi Treatments. Survival curves for *isp-1(qm150); Pgst-4::GFP* animals treated with RNAi targeting the indicated transcription factor. Only extreme survival curves for each RNAi treatment are shown (for important information regarding vector-only RNAi, see Supplementary Figure S3). Descriptive statistics and significance testing for lifespan data is summarized in Table II and Supplementary File 3.

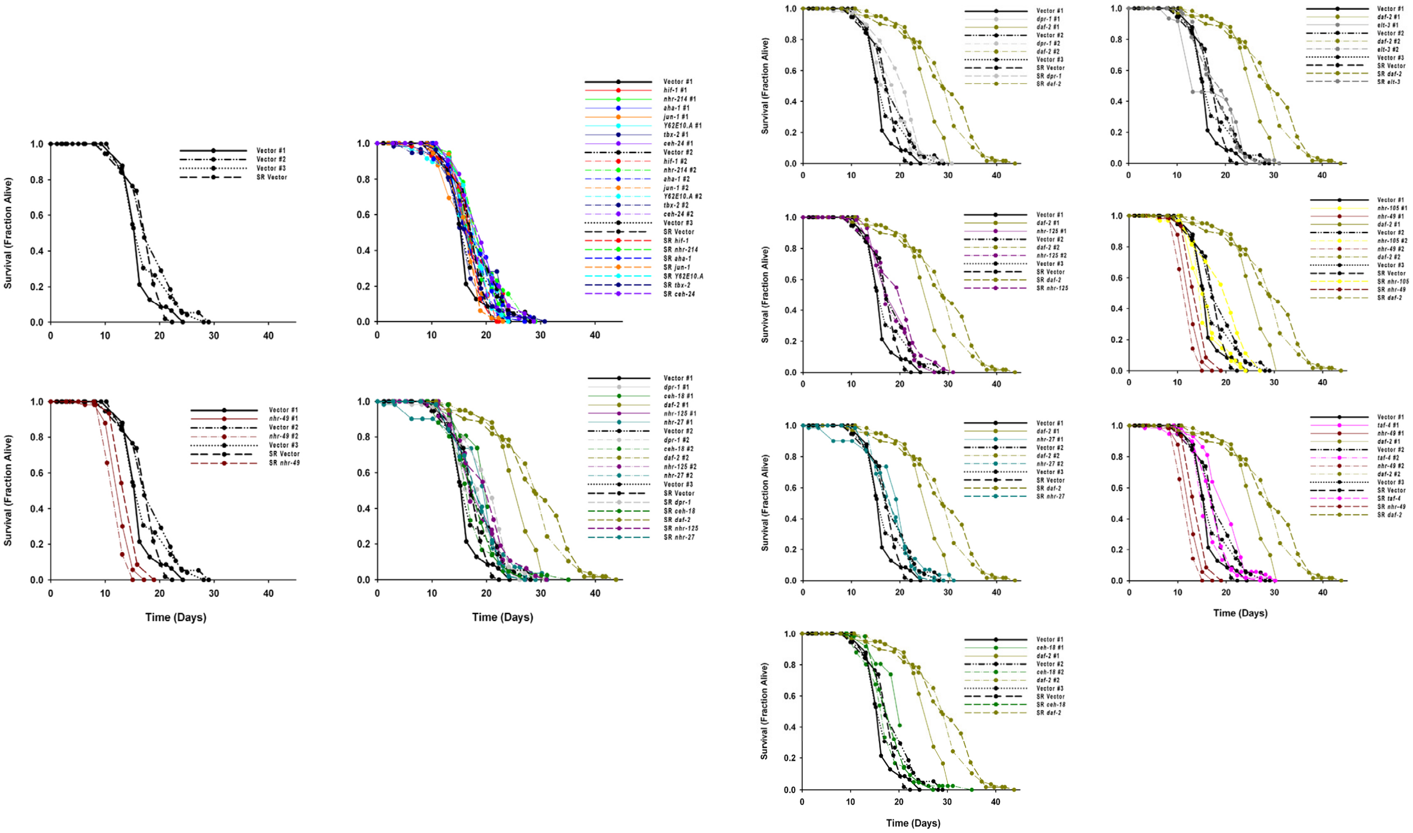

Supplementary Figure S5. *Pgst-4::GFP* Lifespan Studies. Survival curves for *Pgst-4::GFP* animals treated with RNAi targeting the indicated transcription factor. Data are control studies for the *isp-1(qm150); Pgst-4::GFP* experiments shown in Figure 4. Descriptive statistics and significance testing are summarized in Table II and Supplementary File 3.

SUPPLEMENTARY TABLES

Please browse full text version of this manuscript to see the the Supplemental **Table S1** and **S2.**
