# Supplementary material for: TAF-4 is required for the life extension of isp-1, clk-1 and tpk-1 Mit mutants
Source: Aging (Albany NY). 2013 Oct 4;5(10):741–58. doi: 10.18632/aging.100604 (PMC3838777; doi:10.18632/aging.100604)
Supplement: Supplementary file 3 [file aging-05-741-s003.docx]

Summary of TFs Used for Screening

| **Transcription Factor**  **Sub-family** | **wTF2.2**  **(count)** | **Current Study (count)** | **Sub-family Coverage (%)** |
| --- | --- | --- | --- |
| **AP-2** | 4 | 4 | 100 |
| **ARID/BRIGHT** | 3 | 0 | - |
| **AT Hook** | 28 | 0 | - |
| **bHLH** | 41 | 4 | 10 |
| **bZIP** | 31 | 1 | 3 |
| **CBF** | 9 | 1 | 11 |
| **COLD BOX** | 5 | 0 | - |
| **CP2** | 1 | 0 | - |
| **GC-rich DNA-binding domain** | 1 | 0 | - |
| **HD** | 98 | 75 | 77 |
| **HD-like** | 1 | 0 | - |
| **HMG box** | 15 | 3 | 20 |
| **HTH** | 2 | 0 | - |
| **IPT/TIG** | 3 | 0 | - |
| **MADF** | 9 | 0 | - |
| **MADS box** | 2 | 2 | 100 |
| **MH1** | 7 | 0 | - |
| **MYB** | 13 | 1 | 8 |
| **p53** | 3 | 1 | 33 |
| **p66 family** | 1 | 0 | - |
| **Paired Domain** | 7 | 2 | 29 |
| **PUR** | 2 | 0 | - |
| **RNT** | 1 | 0 | - |
| **RPEL - 2 domains** | 1 | 0 | - |
| **SAND** | 2 | 0 | - |
| **STAT** | 1 | 1 | 100 |
| **T-box** | 22 | 16 | 73 |
| **TEA/ATTS** | 1 | 1 | 100 |
| **TESMIN x2** | 1 | 0 | - |
| **TRAP230 family** | 1 | 0 | - |
| **TSC-22/dip/bun** | 3 | 1 | 33 |
| **Winged Helix** | 36 | 26 | 72 |
| **YL1 TF** | 1 | 0 | - |
| **Zinc Finger** | 581 | 247 | 43 |
|  |  |  |  |
| **Total** | **937** | **386** |  |

**Supplementary Table II. Worm Transcription Factor Library.** Summary of the different transcription factor sub-families covered in our study in comparison to all known *C. elegans* transcription factors (as compiled by Reece-Hoyes *et al.* in Worm Transcription Factor version 2.2 (wTF2.2) [[24](#_ENREF_24)])
